# The autopoiesis and the mindfulness as a tool for selfmanagement in health. A theoretical support for a clinical study in psychoneuroimmunology

**DOI:** 10.1192/j.eurpsy.2021.2020

**Published:** 2021-08-13

**Authors:** J.T. Saavedra Perez De Arce, C. Zarate

**Affiliations:** 1 School Of Medicine, Department Of Psychiatry, Universidad San Sebastián, Santiago, Chile; 2 Psychiatry, Clínica Psiquiátrica Universitaria, Universidad de Chile, Santiago, Chile

**Keywords:** Psychoneuroimmunology, mindfulness, Selfmanagement, Autopoiesis

## Abstract

**Introduction:**

Clinical studies had shown a correlation between mindfulness and changes in the immune response. Other studies had observed an interaction between sensory neurons and neuropeptide-mediated immune response.

**Objectives:**

This research aims to provide theoretical support to carry out a clinical study based on psychoneuroimmunology.

**Methods:**

For this, An epistemological analysis of the concepts of autopoiesis and evocative body was carried out to explain the self-conformation of the organism.

**Results:**

The result of this analysis indicates that the autopoietic process of the organism can be experienced from the three levels proposed by the concept of the evocative body (preontological, ontological and logical). It is posible to generate a nexus between the preontological and the logical in the autopoietic process through the ontological level. Mindfulness is the tool through which it is possible to access the ontological and thus express the preontological in the logical, thereby generating the theoretical possibility of being able to influence our therapeutic process.

**Conclusions:**

This analysis supports the concept of the self-management in health as a measurable therapeutic tool in a clinical study.

**Disclosure:**

No significant relationships.

